# ESHG PPPC Comments on postmortem use of genetic data for research purposes

**DOI:** 10.1038/s41431-019-0525-z

**Published:** 2019-10-08

**Authors:** Florence Fellmann, Emmanuelle Rial-Sebbag, Christine Patch, Sabine Hentze, Vigdis Stefandottir, Álvaro Mendes, Carla G. van El, Martina C. Cornel, Francesca Forzano, on behalf of the Public and Professional Committee (PPPC) of the European Society of Human Genetics (ESHG)

**Affiliations:** 1 The ColLaboratory, University of Lausanne, Lausanne, Switzerland; 2 UMR 1027, Inserm, Université Paul Sabatier-Toulouse III, Toulouse Cedex, France; 3 Genomics England, Queen Mary University of London, London, United Kingdom; 4 Society and Ethics Research Group, Wellcome Genome Campus, Hinxton, United Kingdom; 5 Human Genetics, Heidelberg, Germany; 6 Department of Genetics and Molecular Medicine, Landspitali University Hospital, Reykjavik, Iceland; 7 UnIGENe and CGPP—Centre for Predictive and Preventive Genetics, IBMC—Institute for Molecular and Cell Biology, i3S—Instituto de Investigação e Inovação em Saúde, Universidade do Porto, Porto, Portugal; 8 Section Community Genetics, Department of Clinical Genetics and Amsterdam Public Health research institute, Amsterdam UMC, Vrije Universiteit Amsterdam, Amsterdam, The Netherlands; 9 Clinical Genetics Department, Guy’s & St Thomas’ NHS Foundation Trust, London, United Kingdom

**Keywords:** Medical ethics, Ethics

A large number of biobanks are constituted worldwide for many different research purposes. The number of stored samples is increasing, representing a significant proportion of the population in some countries. There is a time lag between sample collection and any potential analysis. Some biobanks aim to collect samples of individuals affected with specific disorders, which can be associated with early death. It is therefore evident that a proportion of samples in biobanks will have been collected from individuals who will be deceased or whose circumstances have changed at the time potential results from analyses are generated. The researchers or biobanks curators are not informed of the death of participants in the vast majority of (if not all) cases. Therefore, researchers proceed with the contribution of those samples without making a distinction between “still alive” and “deceased”. The continuing use of samples postmortem is more implicit than clearly expressed in the current regulations.

In parallel, there are active discussions regarding the return of ‘results’ from research analyses to research participants, either alive or after their death [1, 2]. These may include genetic information, which may have relevance to family members and maybe shared. Bak et al. [3] raise the not trivial issue pertaining the potential disclosure of research results to relatives after the research participant’s death.

Regarding the use of genetic testing after the death, it is stated by the Additional Protocol to the Convention on Human Rights and Biomedicine concerning Genetic Testing for Health Purposes (Article 15) that an informed consent should be obtained from the individual when alive [4]. However, there are no clear recommendations or guidelines available at present regarding communication to relatives of research (genetics) results obtained postmortem on an individual who consented to participate when alive. As a result, the future storage and use of the samples after the participant’s death is frequently not addressed in the majority of consent forms, as reviewed by Bak et al. [3].

In 2003, the ESHG recommendations on data storage and DNA banking for biomedical research [5] stated: « investigators should be required to recontact subjects to obtain consent for new studies. If it is impracticable to gain consent, an appropriate ethics review board should give its consent for further use of the samples based on the notion of minimum risk for the donor. Concerning postmortem uses of samples, a policy of unrestricted access cannot be justified on the grounds that the risk or harm for the subject are no more an issue. If individuals restrict use of their sample when they are still alive, those restrictions apply after their death. » Decisions should therefore be based on the choices of the participant at recruitment, as stated on the consent form. Where this clause is not present, an Ethical Committee should be consulted on the best way to proceed.

Although the 2003 ESHG document has contemplated this specific situation, and provided some guidance, we agree with Bak et al. that the issue was only tangentially addressed and not thoroughly discussed. Also, changes in practices and introduction of powerful technologies both in research and diagnostic settings might merit a reappraisal.

In the EU, the General Data Protection Regulation (GDPR) took effect in 2018 [6]. It applies to information that relates to an identifiable living individual. Information relating to a deceased person is not subject to the GDPR. However, some national legislations have already adopted some specific provisions with regards to the use of personal data after the death under the umbrella of the fundamental right of privacy allowing individuals to make decision of the future use of their data when they are alive. This position has been recently adopted in France [7]. Interestingly, the Health Insurance Portability and Accountability Act [8], which is the GDPR equivalent in USA, considers that the health information of an individual deceased within the last 50 years is considered as Protected Health information. Health information of individuals deceased more than 50 years is not considered as protected.

Overall, the implications of the continuing (postmortem) use of samples is not specifically addressed in existing guidelines or recommendations. The Institutional Review Boards are responsible to give an authorization for research, as far as individuals did not restrict the use of their samples.

The review from Bak et al. focuses on postmortem use of previously acquired genetic and health-related data for research purposes. It is important to underline that, in practice, the distinction between research and diagnostics is sometimes very narrow particularly in the case of rare diseases.

The ESHG recently published recommendations for recontacting patients in clinical genetics services [9]. However, these recommendations did not specifically address the case of the patient’s death occurring in the interval between the samples being obtained and the result being generated.

The impact of postmortem genetic testing and the feedback of results to relatives have been mentioned in the European recommendations published in 2004 by an expert group invited by the European Commission [10]. The 24th recommendation addresses postmortem genetic analysis, stating that member states are to take action to promote the right of access to samples and data from a deceased person, in the case of the overriding interest of blood relatives.

More recently, the ESHG published recommendations integrating genetic testing into the multidisciplinary management of sudden cardiac death [11]. In these particular cases, samples are taken after the death for diagnostic purposes and the genetic test performed in this context might benefit the deceased’s relatives.

One major difference between research and diagnostic use might be represented by the anonymisation of the samples. However, the feasibility of complete anonymisation remains debated in the context of genetic studies [12]. Moreover, and rather differently from the past, current research projects such as 100,000 Genomes Project in UK, Finnish or Estonian biobanks and many others, are being designed to return genetics results that are relevant to participating individuals, as well as to their relatives. Samples and data can therefore not be (completely) anonymized. In the 100,000 Genomes Project, the results are returned by the National Health Service laboratories and clinics and standard practice relating to return of potentially actionable results to family members is followed.

As progresses in medical and genetic/genomic research continue to accelerate, researchers are exposed to new challenges. If the question of postmortem analysis is not new, the disclosure of research results is. Therefore, participants deserve transparency about what is and what is not being disclosed. We suggest that plans for this should be incorporated into the design of the sample collections.

It seems clear that the discussion is more topical than ever and requires updated professional guidance.
